# Properties and physiological effects of dietary fiber-enriched meat products: a review

**DOI:** 10.3389/fnut.2023.1275341

**Published:** 2023-11-30

**Authors:** Bidyut Prava Mishra, Jyotiprabha Mishra, Biswaranjan Paital, Prasana Kumar Rath, Manoj Kumar Jena, B. V. Vivekananda Reddy, Prasad Kumar Pati, Susen Kumar Panda, Dipak Kumar Sahoo

**Affiliations:** ^1^Department of Livestock Products Technology, College of Veterinary Science and Animal Husbandry, Odisha University of Agriculture and Technology, Bhubaneswar, Odisha, India; ^2^Animal Science Division, Krishi Vigyan Kendra, Sambalpur, Odisha, India; ^3^Redox Regulation Laboratory, Department of Zoology, College of Basic Science and Humanities, Odisha University of Agriculture and Technology, Bhubaneswar, India; ^4^Department of Veterinary Pathology, College of Veterinary Science and Animal Husbandry, Odisha University of Agriculture and Technology, Bhubaneswar, Odisha, India; ^5^Department of Biotechnology, School of Bioengineering and Biosciences, Lovely Professional University, Phagwara, Punjab, India; ^6^Department of Livestock Products Technology, NTR College of Veterinary Science, Gannavaram, India; ^7^College of Veterinary Science and Animal Husbandry, Odisha University of Agriculture and Technology, Bhubaneswar, Odisha, India; ^8^Department of Veterinary Clinical Sciences, College of Veterinary Medicine, Iowa State University, Ames, IA, United States

**Keywords:** dietary fiber physicochemical properties, chemical composition, textural properties, organoleptic qualities, fat replacer, nutrition management, nutraceutical value, food fortification

## Abstract

Meat is a rich source of high biological proteins, vitamins, and minerals, but it is devoid of dietary fiber, an essential non-digestible carbohydrate component such as cellulose, hemicellulose, pectin, lignin, polysaccharides, and oligosaccharides. Dietary fibers are basically obtained from various cereals, legumes, fruits, vegetables, and their by-products and have numerous nutritional, functional, and health-benefiting properties. So, these fibers can be added to meat products to enhance their physicochemical properties, chemical composition, textural properties, and organoleptic qualities, as well as biological activities in controlling various lifestyle ailments such as obesity, certain cancers, type-II diabetes, cardiovascular diseases, and bowel disorders. These dietary fibers can also be used in meat products as an efficient extender/binder/filler to reduce the cost of production by increasing the cooking yield as well as by reducing the lean meat content and also as a fat replacer to minimize unhealthy fat content in the developed meat products. So, growing interest has been observed among meat processors, researchers, and scientists in exploring various new sources of dietary fibers for developing dietary fiber-enriched meat products in recent years. In the present review, various novel sources of dietary fibers, their physiological effects, their use in meat products, and their impact on various physicochemical, functional, and sensory attributes have been focused.

## 1 Introduction

Meat is considered an integral part of human nutrition, with rich sources of protein, essential amino acids, fatty acids, vitamins, and minerals, and it provides energy for growth and is involved in various biochemical, metabolic, and physiological activities (1). There is also a growing demand observed in meat consumption patterns and processed meat products by consumers, which might be due to the combined effect(s) of globalization, industrialization, and increase in per capita income of the people as well as working women population and predominance of the nuclear family in the society (2–4). However, due to some negative perceptions of muscle foods due to their high saturated fatty acid (5) and cholesterol content and their possible health hazard effects (6, 7), consumers prefer to consume meat products containing some additional non-meat ingredients having some specific health benefits (8, 9).

There is no doubt about the health hazardous effects of red and processed meat. Pieces of evidence established the strong and moderate carcinogenic effects of red and processed meat, respectively (8). The effects are primarily observed in the alimentary canal. Although consumption of red and processed meat is moderately responsible for causing oxidative stress and genotoxicity by the produced heterocyclic aromatic amines (10) when cooked under high temperatures (11), a meta-analysis states that a positive correlation exists between red or processed meat consumption and adenomas (preneoplastic lesions) (12). In humans, the cause is attributed to a mutation in the adenomatous polyposis coli (APC) gene or methylation (13). Although the consumption of calcium in diet along with red or processed meat reduces the chance of colorectal cancer (14), more clearly, consumption of red and processed meat leads to lipid oxidation (15), generation of oxidative stress toxicity, cancer as observed in human and rodents bio-samples such as fecal matter, blood, and urine (16–18), and formation of DNA adducts (at a consumption rate of 300 or 420 g day^−1^) by polycyclic aromatic hydrocarbons (19) that are produced under high heat treatment to red and processed meat (20, 21). Overall, processed meats can be “(probably) carcinogenic to humans” as evidentially they cause cancer in the colon (22), colorectal (23–26), pancreatic, and prostate (8). Therefore, a reduction in the cancerous properties or any other health-hazardous properties of red and processed meat, for example, a reduction in the chance of colorectal cancer with the addition of calcium to the diet, is required.

Generally, meat is devoid of dietary fiber, abundantly found in plant materials, and has various physiological activities in the human body. Epidemiological studies reported that the intake of foods with low dietary fiber is one of the major risk factors for the prevalence of many lifestyle diseases (27). Processed foods augmenting disease prevention and/or health benefits, in addition to their nutritional values, are known as functional foods. Functional meat products can be developed either by incorporating some health-promoting non-meat ingredients or by removing some undesirable ingredients present in the meat, such as fat and cholesterol (9, 11, 28, 29). Dietary fibers can act as an excellent meat substitute as well as an efficient fat replacer (30, 31) during the preparation of functional meat products due to their inherent functional, physiological, and nutritional effects (32).

Foods containing high dietary fiber are known to decrease the chances of occurrence of various cancers, diabetes, hyperlipidemia, cardiovascular diseases, obesity, gastrointestinal disorders, inflammatory bowel diseases, and neurological disorders (33). Moreover, a growing interest has been noticed among meat scientists, meat food processors, and researchers in developing various new functional meat products by incorporating dietary fibers from various natural plant sources. So, in the present review, various new sources of dietary fibers, their physiological effects, applications in meat products, and their impact on various physicochemical, functional, and sensory attributes have been discussed.

## 2 Dietary fiber

Dietary fiber is now popularly called a “universal remedy” by food scientists with diverse health-promoting activities (34). The word “dietary fiber” was initially used by Hipsley (35), who opined the non-digestible constituents of the plant cell wall as the dietary fiber. Since then, various researchers have made many revisions regarding the definition of dietary fiber at different times. Trowell (36) defined dietary fiber as those parts of fruits, vegetables, nuts, and whole grains that are digested very poorly by the human digestive system. The term plantix was used by Spiller et al. (37) to denote those undigested plant materials that form a complex matrix in the human digestive tract. Kay (38) designated the term dietary fiber to those parts of plant foods with diverse morphological and chemical structures that resist the action of the enzymes of the human digestive system. Later on, it was defined by Trowell et al. (39) as that portion of the plant cell materials such as lignin, hemicellulose, cellulose, pectin, polysaccharides, gums, oligosaccharides and waxes which remain unaffected to the hydrolytic action of the endogenous enzymes of the human alimentary tract.

As per the American Association of Cereal Chemists (AACC) in the year 2000, it is defined as leftover of the edible parts of plants or similar carbohydrates that resist their digestion and absorption in the small intestine of human beings and ferment partially or completely in the large intestine. Australia New Zealand Food Authority in the year 2001 defined dietary fiber as the eatable parts of plant materials or their extracts, or other similar carbohydrates, which remain refractory to digestion and absorption in the small intestine with partial or complete fermentation in the large intestine of human beings. In the year 2002, the National Academy of Science used the term dietary fiber complex to denote the combination of both dietary fiber, which are indigestible carbohydrates, and lignin, which are fundamental to plants, and functional fibers, which are isolated and non-digestible carbohydrates having health benefits in human beings. Dietary fibers remain resistant to the action of endogenous digestive enzymes in the upper digestive tract of humans and are not absorbed and utilized in the body (40). The natural sources of dietary fiber are various cereals, legumes, fruits, nuts, and vegetables. Generally, cereals, brans, and husks are considered the major sources of cellulose, lignin, and hemicellulose, whereas fruits and vegetables are considered the principal source of mucilage, pectin, and gums (41, 42) and leafy vegetables as the source of lignin in the diet (43).

Dietary fibers are classified as non-starch polysaccharides, resistant oligosaccharides, resistant starch, and lignin based on their chemical properties (44). Based on their source, they can be classed into plant-based polysaccharides, animal-origin polysaccharides, and synthetic forms (45). Most commonly, dietary fibers have been divided into two types, such as soluble dietary fibers (SDF) and insoluble dietary fibers (IDF), based on their water solubility (40). Primarily, lignin, cellulose, and part of hemicelluloses are considered insoluble dietary fibers, whereas pectins, pentosans, β-glucans, gums, mucilages, and various types of non-digestible oligosaccharides along with inulin are known as source of soluble dietary fibers (45–47). Soluble fibers are again of two types, viscous and non-viscous fibers, which are always fermentable. The insoluble fibers are always non-viscous and are usually poorly fermented or non-fermentable in nature. Each type of fiber has different physiological functions inside the body.

**Figure 1:**
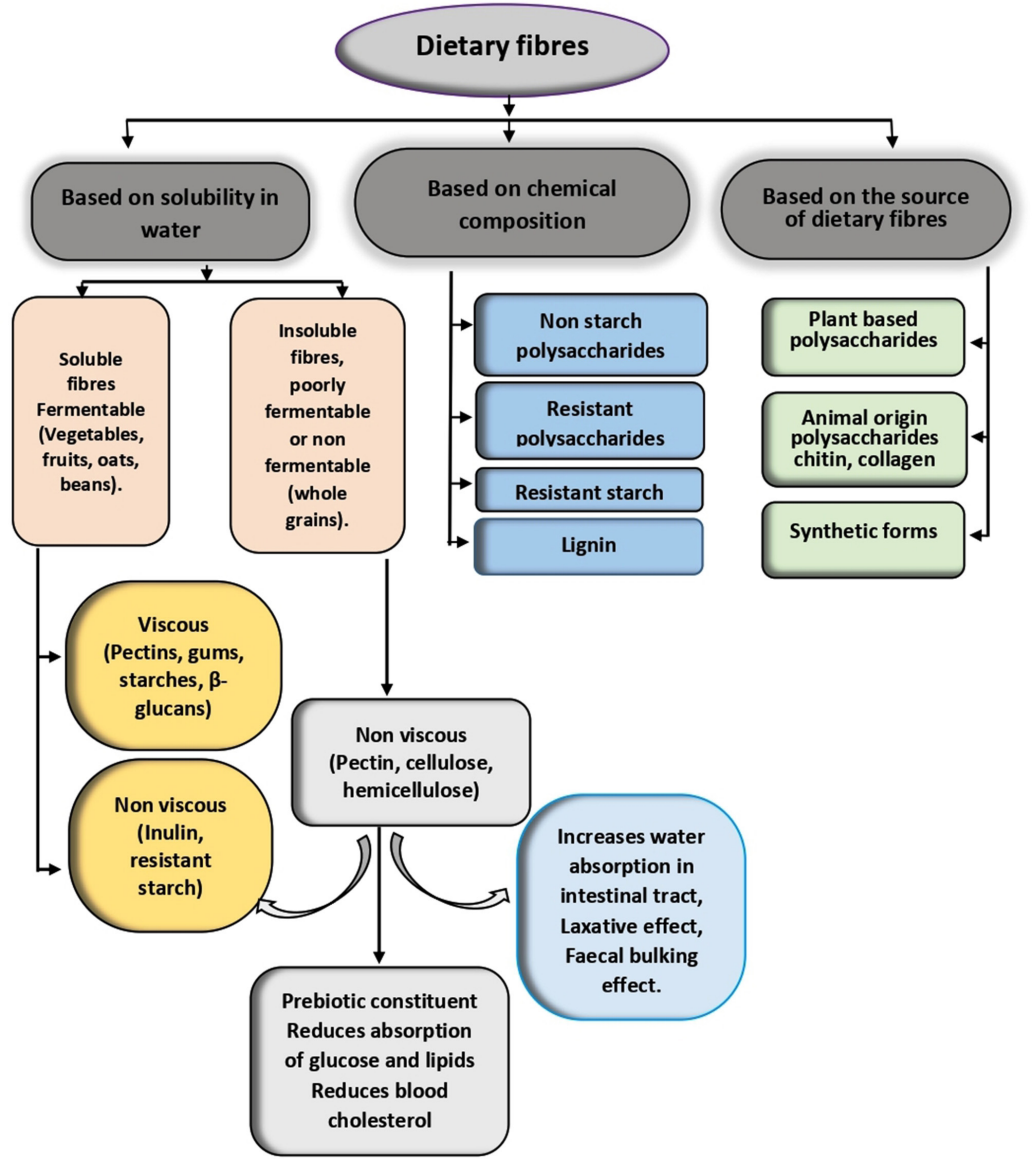
Classification of dietary fibers.

Soluble dietary fibers are mainly responsible for reducing blood cholesterol and reducing the absorption of glucose in the small intestine and act as a potential prebiotic constituent (48, 49), whereas insoluble fibers increase water absorption in the intestinal tract and regulate the other intestinal activities (50). The insoluble fibers are more common in foods than the soluble fibers. Usually, oats, oat brans, rice, barley, peanuts, peas, lentils, black beans, kidney beans, papaya, banana, pears, apricots, dried figs, mangoes, oranges, avocado, flax seeds, pumpkins, carrots, etc. are considered as the major sources of soluble dietary fibers in the nature (51), whereas apples, sprouts, wheat flour, wheat bran, dates, green leafy vegetables, pineapple, cabbage, cauliflower, broccoli, nuts, whole grains (52, 53), etc. are considered as the principal source of insoluble fibers. The classification of dietary fibers is presented in Figure 1.

## 3 Physiological effects associated with dietary fibers

**Figure 2:**
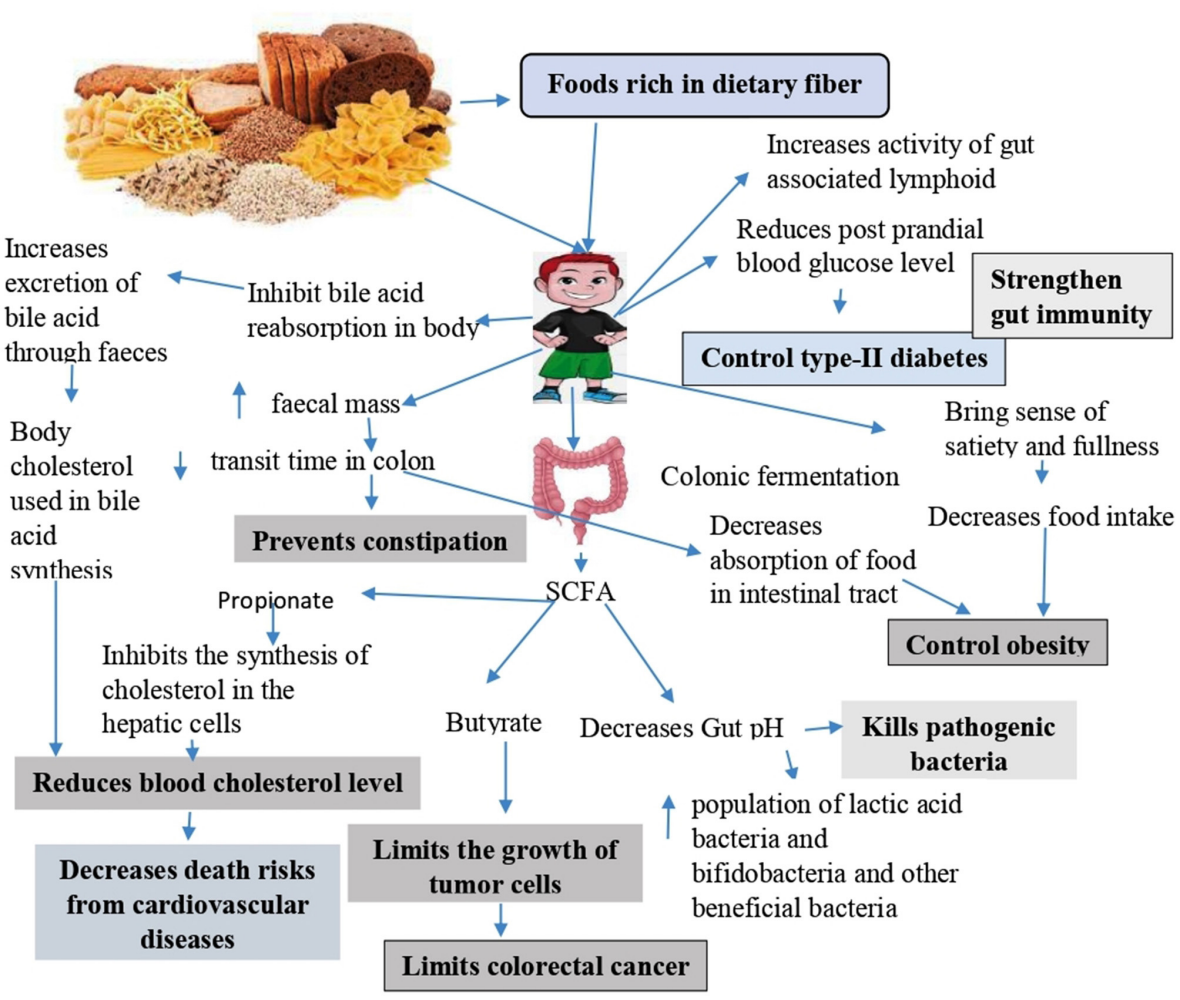
Physiological health benefits associated with dietary fibers.

Dietary fibers play a very crucial role in controlling and preventing many lifestyle diseases. Each fiber has its unique protective mechanism based on the type of fiber and its composition. Health benefits associated with dietary fiber are presented in Figure 2.

### 3.1 Prevention and control of cardiovascular diseases

Regular consumption of the recommended level of dietary fiber reduces the death risks from strokes and cardiovascular diseases (54) by lowering the blood cholesterol level. The mechanism behind dietary-induced elevation of heart health is explained in many biochemical and molecular pathways. Dietary meals supplemented with oatmeals, nuts, fruits, citrus, pears, etc. increases the titer of blood high-density lipoprotein (HDL), which clears the stiffness in blood vessels by removing the deposited low-density lipids (55). Low-density lipoproteins (LDLs) and triglycerides usually act as the master molecules for blocking the arteries. The blood HDL removes the low-density lipoprotein (LDL) from arteries back to the liver where they are metabolized (56). In turn, it reduces cholesterol-mediated heart health issues, especially cardio-arterial blockages and cardiac stroke (57). So, high dietary fibers in turn reduce the chance of cardiac attack and maintain normal arterial pressure (58).

High HDL also increases arterial stiffness, which indirectly decreases insulin resistance in humans (55, 59). The mechanism is attributed to the triglyceride glucose index and triglyceride/HDL–cholesterol ratio level in the blood. Elevated ratio modulates brachial-ankle pulse wave velocity that is mainly linked to the arterial stiffness progression in hypertensive populations. However, such phenomenon is not observed in the prehypertensive population (55). It indicates that the stiffness in arteries maintained by HDL is beneficial by decreasing insulin resistance in human samples. This in turn regulates heart health on the one hand and the hyperglycemic condition on the other hand (60).

Some specific dietary fibers such as guar gum, β-glucan, psyllium, and pectin inhibit the bile acid reabsorption in the body (61) and increase its excretion through feces (62). Cholesterol is converted into bile acids. Along with cholesterol, bile and phospholipids produce mixed micelles to solubilize further cholesterol. Through this process, bile acids are utilized and are not allowed for reabsorption. So, on one hand, bile acid synthesis occurs at the expense of cholesterol present in the body, and on the other hand, it indirectly helps in reducing the blood cholesterol level (63).

High dietary fiber intake is associated with reducing premature mortality rates in patients with pre-existing cardiovascular diseases and hypertension by lowering the total and low-density lipoprotein cholesterol and reducing the systolic and diastolic blood pressure (27). Regular fiber intake along with food also helps in providing additional antioxidants, decreasing the role of oxidative stress factors in the occurrence of atherosclerosis (64) and enhances the pliability of the blood vessel wall to lower vascular resistance and keep enough tissue perfusion without needing a subsequent increase in heart rate to keep stroke volume (27). Other beneficial elements included in high-fiber meals, such as vegetables, are metabolized into compounds such as nitric oxide, which may lower blood pressure by increasing its bioavailability for usage in vasodilation (65). Additionally, it has been proposed that the short-chain fatty acids produced during fiber fermentation may possibly play a role in mediating the hypocholesterolemic action of dietary fibers. According to reports, propionate helps reduce the blood cholesterol level by inhibiting the synthesis of cholesterol in the hepatic cells. The viscous qualities of soluble fibers may prevent cholesterol absorption, and glucose and fiber viscosity have also been suggested to enhance glycemic management and cholesterol concentrations.

### 3.2 Effect on control of diabetes

Many studies reported that a diet with low fiber content and high glycemic index causes the individual to develop type 2 diabetes (66). It is suggested that dietary fibers of some whole grain foods help in reducing the post-prandial blood glucose level, which lowers insulin requirements and prevents the pancreas from becoming overworked and thus controls diabetes (67). Due to their high water-holding capacity, ingested fibers impede stomach emptying by producing a gel matrix (68), and this matrix may thicken intestinal contents and reduce the interaction between food and digestive enzymes, slowing down the digestion and absorption of carbohydrates (69), thus controlling diabetes.

### 3.3 Role in control of obesity

Due to less energy density and the high voluminous nature of the fibers, they bring a sense of satiety and fullness. Inclusion and consumption of high-fiber diet in a regular meal decreases the volume of the rest of the meal, and thus control the food intake, which in turn decreases the chance of obesity in human beings (70). More intake of dietary fiber controls blood sugar levels because the tissues are disallowed to absorb and break down fiber; as a result, it does not spike blood sugar levels as the carbohydrate diets do. High dietary fibers also modulate insulin resistance, thus controlling blood sugar levels in the allowed range. This in turn controls fat deposition and obesity in the body (71, 72).

More intake of dietary fiber lowers insulin secretion, which helps in mobilizing and utilizing fat depots (73). The body weight loss rate is directly proportional to the consumption level of dietary fiber (74) and dietary fiber type (70). It helps in rapid gastric emptying by reducing the transit time of digested food in the gastrointestinal tract, thus lowering the absorption of food in the body.

### 3.4 Role of dietary fiber in controlling cancer

Dietary supplementation with functional nutraceuticals has proven to be beneficial for the human body (75, 76). The role of dietary fibers in protection from colon, breast, and prostate cancers has been reviewed (77). Statistics indicate from the European and American populations that high-fiber intake in diets reduces the risk of colon cancer. Between the year 1980 and 1981, 32 studies were performed to find out the association of dietary fiber and cancer. Of the 32 studies, 25 confirmed the inverse relation between dietary fiber and cancer risk globally (78). Similarly, a meta-analysis performed in 2018 to find out the relationship between cancer risk and low fiber diet, considering results from PubMed between the years 1980 and 2017, indicates that statistically significant reduction in the relative risk of colorectal, esophageal, gastric, and pancreatic cancer was 0.52–0.88, whereas for the breast cancer, the reduction value of relative risk was 0.85–0.93. The cancer mortality rate can be reduced by 17% with a recommended dietary fiber content of 38 g per day (79). Recently, another systematic review also concluded that dietary fiber intake induces different molecular and biochemical pathways to prevent the risk of cancers (80, 81).

Therefore, it is indeed an accepted scientific fact that dietary fiber plays an important role in the prevention and control of colon cancer, either by preventing interactions between cancer-causing agents and the intestinal mucosa by increasing fecal mass (82) or by inhibiting the production of carcinogenic constituents in the colon (83). Moreover, the SCFAs produced in the large intestine during the fermentation of a fiber-rich diet plays a major role in reducing the incidence of colorectal cancer (84). It has been reported that butyric acid prevents malignancy in cells by reducing the production level of secondary bile acids in acidic pH and increasing cell proliferation (85). In addition, some dietary fibers also act as antioxidative agents in the human body and strengthen the individual's immune system (32). Some studies also reported the protective action of fibers in the control of breast cancer by increasing the losses of estrogens through fecal masses, which is a potent cause for the occurrence of breast cancer.

### 3.5 Fiber and maintenance of gut microbiota

Dietary fibers remain resistant to the digestive action of enzymes present in the small intestine of human beings. They usually get fermented in the large intestine with the help of colonic microflora to produce various short-chain fatty acids (SCFAs) having health-benefiting effects such as acetate, butyrate, and propionate along with carbon dioxide, hydrogen, and methane. The pH in the gut becomes acidic in the presence of SCFAs, which inhibits the growth of pathogenic bacteria and helps in the proliferation of beneficial bacteria such as lactic acid bacteria and bifidobacteria (86). Dietary fibers increase the colonic microflora and fecal mass and reduce the transit period in the colon, thus preventing constipation. Some studies evidenced that fermentable dietary fiber causes an increase in the T cell mitogen response and the activity of gut-associated lymphoid tissue, which helps in maintaining gut immunity (87).

## 4 Functional characteristics of dietary fiber

Fiber inclusion in meat products is gaining more importance for its numerous functional as well as technological properties such as water retention, lubrication, freeze/thaw stability, fat binding ability, gel-forming capacity, texture modification, neutral flavor, ability to decrease cooking loss (33, 88), and disease preventing abilities. Many dietary fibers from various plant sources, whole grains, fruits, cereal fibers, and vegetables have been used successfully in meat formulations with proven efficiency (45). Dietary fibers from fruit and vegetable sources having better oil binding ability are used to prepare mostly emulsion-based processed meat products (34, 45). The chemical composition, structure, type of fibers (89), ionic strength, pH, and particle size (90) greatly influence the water-holding capacity (WHC) and oil-binding ability of the dietary fibers (45, 88). Meat products incorporated with dietary fiber increase the emulsion stability (91), yield of the product, textural characteristics, water-binding capacity in minced meat, replace the fat (31, 91), maintain shape in the heat-treated products, and increases storage stability by stabilizing proteins and fats. A list of fibers utilized in the preparation of various dietary fiber-enriched meat products has been mentioned in Table 1.

**Table 1:** List of dietary fiber utilized in the preparation of various fiber-enriched meat products.

**Source of dietary fiber**	**Processed meat product**	**References**
Cactus, pear, and pineapple fibers	Sausages	(92)
Cellulose fibers	Chicken meatballs	(93)
Hemicellulose B	Meatballs	(94)
Carboxymethyl cellulose	Chicken meatballs	–
	Beef patties	(95)
Chickpea flour	Beef patties	(96)
Wet okara and Okara powder	Beef patties	(97, 98)
Inulin	Chicken meatballs	(93)
	Chopped cooked chicken products	(99)
	Pork loaves	(100)
	Emulsion type sausages	(30)
	Beef burger	(101)
Wheat bran	Chicken meat patties	(102)
	Chicken sausages	(103)
Wheat flour	Beef patties	(96)
Wheat fiber	Dry fermented sausage	(104)
	Beef burger	(101)
Oat fiber	Beef burger	(101)
	Dry fermented sausage	(104)
Oat flour	Beef patties	(96)
Oat bran	Chicken meat patties	(102)
	Meatballs	(105)
Rice bran	Frankfurters	(106)
	Frankfurters	(107)
	Hamburger	(31)
	Chicken meat rolls and patties	(108)
Rice flour	Beef patties	(96)
	Dehydrated chicken meat rings	(109, 110)
Rye bran	Meatballs	(111)
Psyllium husk	Chicken meat rolls and patties	(108)
Aloe vera	Nuggets	(112)
Chia flour	Chicken nuggets	(113)
Pea flour	Bologna	(114)
Chickpea flour	Beef patties	(96)
Corn flours	Beef patties	(96)
Corn bran	Chicken sausages	(115)
Barley flour and yellow lentil flours	Beef patties	(96)
Soy flour	Beef patties	(96)
Barnyard millet flour	Dehydrated chicken meat rings	(116)
Jerusalem artichoke powder	Sausage	(117)
Jabuticaba skin	Restructured hams	(118)
Fructooligosaccharide	Low-fat beef burger	(101)
Lotus rhizome	Sausage	(119)
*Algelica keiskei* koidz fiber	Chicken patties	(107)
Guar gum, xanthan gum, and gum arabic	Beef patties	(120)
Maize	Turkish meatballs	(121)
Dried carrot pomace	Chicken sausages	(103)
Black gram hull	Chicken meat rolls and patties	(122)
Tomato fiber, beetroot fiber	Chopped cooked chicken products	(99)
Dried tomato pomace	Chicken sausages	(115)
Pumpkin fibers	Frankfurters	(91)
Dried apple pomace	Chicken sausages	(123)
Kinnow pomace powder	Pork patties	(124)
Peach fiber, apple fiber, and orange fiber	Dry fermented sausage	(104)
Carrot fibers	Chicken meatballs	(93)
Citrus fiber and collagen	Frankfurters	(106)

### 4.1 Effect of dietary fiber on the physicochemical properties of the meat products

The physicochemical properties such as pH, cooking yield, and water-binding capacity of the meat products are varied when they are fortified with dietary fibers. So, looking into these perspectives is essential for updating knowledge on this aspect.

The techno-functional characteristics, as well as the storage stability of meat products, are greatly influenced by the pH of the meat. The ultimate pH of the meat after completion of rigor mortis in slaughtered animals usually falls in the range of 5.4–5.6. The pH in meat products is generally altered by cooking meat, the effect of added ingredients in it, or the storage conditions. The pH in fiber-added products is generally influenced by the pH of the fiber used, the type or source of the fiber, and the amount of dietary fiber added.

Turhan et al. (97) reported an increased pH value in okara powder-added beef patties in both raw and cooked patties. However, the authors also noted that the pH value in cooked patties was higher than in raw patties. The mean pH value of raw beef patties with okara powder at different levels (0, 2.5, 5, and 7.5%) ranged from 5.16 to 5.30, whereas the value for cooked patties ranged from 5.35 to 5.57, indicating the influence of cooking on pH. Barnyard millet (*Echinochloa frumentacaea*) flour is a rich source of dietary fiber (9.8 g/100 g of edible portion), calcium, phosphorous, magnesium, and manganese (116). Mishra et al. (109, 110) utilized rice flour (RF) and barnyard millet flour (BYMF) in the development of dehydrated chicken meat rings and investigated their effect on the physicochemical properties. The authors reported a lower dehydration ratio and higher yield percentage in RF and BYMF-added meat rings than the control. Yadav et al. (103) utilized dried carrot pomace and wheat bran in chicken sausage formulation and observed that the pH of the sausages with 0%, 3%, 6%, and 9% of wheat bran ranged from 6.28 to 6.36 and increased significantly with an increase in the level of wheat bran, whereas the pH of sausages with 0%, 3%, 6%, and 9% of dried carrot pomace ranged from 6.28 to 5.96 and decreased significantly with increase in the level of dried carrot pomace.

In another study, Yadav et al. (115) studied the effect of the addition of dried tomato pomace, corn bran, and dried apple pomace at three different levels (3%, 6%, and 9%) on the pH of chicken sausage and noted no significant difference in pH in corn bran added products, whereas pH decreased significantly in sausages with added dried apple pomace as well as dried tomato pomace with increase in level of incorporation of fibers. This variation in pH was attributed to the different pH values of the fibers used. It is reported that the mean pH values of dried apple pomace and corn bran were 4.59 and 5.97, respectively. Kumar et al. (124) incorporated dried kinnow pomace powder in pork patties and observed that the pH decreased significantly with an increase in the level of kinnow powder in the patties, and this decrease was attributed to the acidic pH value of the fiber, i.e., 3.40.

Cooking yield and emulsion stability (ES) are two other important quality attributes of meat products as they are directly associated with decreased cost of production by increasing the production figure. Soluble dietary fibers are generally associated with high water-holding ability and high oil-binding capacity. These properties help increase the cooking yield when added to the meat products during processing (33). Fiber source and quantity of fiber also influence the cooking yield as well as the ES of the meat product. Moreover, WHC is another quality attribute of the meat product, which plays a major role in developing positive quality characteristics in processed meat products. WHC in cooked meat products can be enhanced by incorporating dietary fibers due to their inherent higher water-binding abilities.

Yasarlar et al. (121) noted that weight loss in Turkish meatballs decreased with increased levels of wheat, oat, rye, and corn bran (5%, 10%, 15%, and 20%, respectively). Mehta et al. (108) investigated the effects of psyllium on the physicochemical properties of chicken patties. The cooking yield and ES increased as the amount of husk increased. They attributed the increase to the presence of a higher amount of SDF entrapping and holding moisture in the form of a gel during the application of heat. A decline in cholesterol content and increased total dietary fiber (TDF) and SDF of chicken patties with psyllium husk were reported. Mean TDF and SDF values ranged from 0.33 to 2.95% and 0.16 to 2.77%, respectively. The addition of *Algelica keiskei* Koidz dietary fiber in chicken patties resulted in a significant decrease in cooking losses and a reduction in diameter and thickness compared to the control product (125). The authors attributed the decrease to the higher water-holding and water-binding abilities of *A. keiskei* koidz dietary fiber, which holds the meat tissues together during heating by forming a stable complex and preventing the deformation of the shape. Cooking yield, ES, moisture retention, fat retention, and muscle:protein ratio parameters of chevon patties increased with increasing finger millet flour levels (2%, 4%, and 6%) (126).

The improvement in ES and cooking yield was attributed to the higher water-binding ability of fibers present in finger millet flour and the retention of fat in the cooked product. The quality of the meat patties mostly depends on their dimensional characteristics, such as changes in diameter and thickness or height of the product. The shrinkage% decreased, and the percent gain in height increased with the increased levels (2%, 4%, and 6%) of finger millet flour in chevon patties. This improvement was attributed to higher WHC and better moisture retention ability of the fibers present in the product. The higher fat retention in chevon patties with added finger millet flour was attributed to the embedment of fat globules in the gel structure of the protein–starch network formed by the starch component of the finger millet flour during cooking. Adding carrot powder to chicken cutlets resulted in more moisture due to better WHC of the fibers present in the carrot (127). The mean moisture value ranged from 58.80 to 61.05%. Similar findings with respect to improved WHC resulting in higher moisture content have been outlined earlier (128, 129).

Kim et al. (91) studied the effects of minimizing fat levels from 30% to 25%, 20%, and 15% by substituting pumpkin fiber (2%) with pork fat and water in the frankfurters with respect to some physicochemical properties. Pumpkin fiber at a 2% level decreased the water and fat exudation of reduced-fat meat batter compared to the control prepared without pumpkin fiber. ES and cooking yield were enhanced by incorporating dried tomato pomace, corn bran, and dried apple pomace at 6 and 9% levels in the chicken sausages (115). Kilincceker and Yilmaz (120) studied the effect of the addition of guar gum, xanthan gum, and gum arabic at 0.5%, 1%, and 1.5% levels on the physicochemical properties of the beef patties. They reported an increased frying yield and decreased diameter reduction percent in patties containing 1 and 1.5% guar gum and xanthan gum, respectively. Increased moisture retention was observed in all types of gum-added beef patties at 1 and 1.5% levels. The cooking yield and ES of chicken sausages increased significantly at a 6% level of fiber incorporation. This improvement was assigned to increased water retention by adding wheat bran and dried carrot pomace (103). Kilinççeker and Kurt (93) utilized inulin, carrot, and cellulose fibers at three different levels (3%, 6%, and 9%) in chicken meatballs. They reported that frying yield decreased with increasing levels of inulin in meatballs, and dimension reduction in fried meatballs was absent in carrot-added meatballs. Moisture retention decreased with increasing levels of inulin and at 9% level of carrot-added meatballs. The fat absorption value of meatballs increased with increasing levels of carrot and cellulose fibers.

### 4.2 Effect of dietary fiber on the chemical composition of meat products

The binders, extenders, or fillers used in the formulation of various meat products tend to alter the composition of the developed product as the chemical composition of those ingredients is completely different from the composition of meat. The chemical composition of fiber-added meat products is greatly influenced by the type of fiber source used and its proportionate level in the product. The recent research findings on the effect of dietary fiber on the chemical composition of meat products have been critically reviewed and mentioned in Table 2.

**Table 2:** Effect of the addition of dietary fiber on the physicochemical and chemical composition of meat products.

**Source of dietary fiber**	**Meat product**	**Optimum level of incorporation by replacing meat**	**Changes in attributes**	**References**
Oat bran	Meatballs	20%	Fat and moisture, total fat, and total *trans* fatty acids content decreased Ash and protein contents increased	(105)
	Chicken meat patties	10 and 15%	Moisture, protein, fat, and cholesterol contents decreased Water-holding capacity, emulsion stability, cooking yield, firmness, total dietary fiber, and unsaturated fatty acids increased	(102)
Rye bran	Meatballs	5 and 10%	Fat and moisture contents decreased Ash and protein contents increased lower concentrations of total fat and total *trans* fatty acids	(111)
Wheat bran	Meatballs	20%	Fat, moisture content, and weight loss decreased Ash and protein contents increased Total fat and total *trans* fatty acid contents decreased	(130)
Oat flour	Beef patties	4%	Moisture decreased in raw patties Moisture increased in cooked patties Cooking yield decreased	(131)
Rice bran	Pork meatball	< 10%	White index, protein, and fat contents decreased	(132)
	Hamburger	4%	Moisture content increased Fat content decreased Cooking loss decreased	(31)
Cereal brans (oat, maize, rye and wheat)	Turkish meatballs	10%	Fat and moisture contents decreased Fiber, ash, and protein contents increased Weight loss decreased	(121)
Okara powder	Beef patties	7.5%	Moisture and cholesterol contents, cooking loss percent, and reduction in diameter percent decreased Fat, ash, carbohydrate content, pH, and WHC value increased	(97)
Oat fiber and wheat fiber	Chinese-style sausages	3.5%	Moisture content decreased No significant difference in protein, fat, ash, and carbohydrate contents	(133)
Inulin, tomato fiber and beet root fiber	Chicken batter	3%	pH value decreased WHC increased	(99)
Psyllium	Chicken patties		Cooking yield and emulsion stability increased Cholesterol content decreased Total dietary fiber increased	(108)
Finger millet flour	Chevon patties	6%	Moisture, carbohydrate, and ash contents increased Fat and protein contents decreased Moisture-protein ratio and fat retention increased Cooking yield, emulsion stability, moisture retention, fat retention, muscle: protein ratio increased shrinkage% decreased% gain in height increased	(126)
*Aalgelica keiskei* koidz	Chicken patties	2%	Moisture and ash contents increased Energy value decreased Cooking losses decreased, reduction in diameter and thickness	(125)
Carrot powder	Chicken meat cutlets	4%	Moisture, ash, and crude fiber contents increased Fat and protein contents decreased	(127)
Barnyard millet flour	10%	Dehydrated chicken meat rings	Moisture, protein, fat, ash, total lipids, phospholipids and cholesterol contents decreased Iron and manganese contents increased Higher yield Lower dehydration ratio	(109, 110)
Dried apple pomace	Chevon roll	6%	Lowered protein and moisture contents	(134)
	Chicken sausage	6%	Increased crude fiber content and decreased moisture content, whereas protein content and pH decreased	(115)
Aloe vera gel	Goat meat nuggets	2.5%	Moisture content increased Protein content decreased	(112)
Gum arabic	Fried beef patties	1.5%	Increased moisture content and decreased fat content Frying yield Increased Diameter reduction percent decreased	(120)
Inulin powder	Pork loaves	2%	Cooking yield, emulsion stability, fat retention, and crude fiber contents increased, and calorific value decreased	(100)
Lotus (*Nelumbo nucifera*) rhizome powder	Cooked sausages	3%	Moisture content and cooking losses decreased Insignificant difference in pH Emulsion stability and apparent viscosity increased	(119)
Dried carrot pomace	Chicken sausage	6%	Protein, fat, and moisture content decreased Ash, crude fiber content, cooking yield, and emulsion stability increased	(103)
Fructooligosaccharide	Beef burger	6%	Moisture content decreased Carbohydrate content increased No change in ash, protein, and fat contents pH value decreased	(101)
	Chicken meatballs	3%	Yield, moisture absorption, and diameter reduction values increased	(93)
Dried kinnow pomace powder	Pork patties	4%	Moisture, protein, fat content, and pH decreased Crude fiber and ash contents increased Cooking yield and emulsion stability increased	(124)

Oat bran and oat flour are the best-known sources of soluble dietary fibers. They help to lower the serum cholesterol level and the risk of cardiovascular diseases. They also help to reduce the absorption of fat and carbohydrates in the human gastrointestinal tract and aid satiety (135). Wheat bran, rye bran, rice bran, and most other grains are considered good sources of insoluble dietary fiber (136). Wheat brans prevent and control many bowel disorders and cancers (111). The addition of wheat bran in low-fat products helps in retaining the added water due to their high WHC. The consumption of rye helps to inhibit colon and breast tumors in animal models, lowering the risk of diabetes and cardiovascular diseases (137).

Huang et al. (133) studied the use of inulin, wheat fiber, and oat fiber in Chinese-style sausages and found that the addition of fibers decreased the moisture content in the sausages and no significant difference for protein, fat, ash, and carbohydrate contents. Crude fiber content in sausages increased with the addition of oat fiber and wheat fiber. The mean crude fiber value ranged from 0.04 to 3.89%. They reported that the water retention value, oil retention value, and water solubility value of wheat fiber were 5.88 ml/g, 4.98 ml/g, and 4.2%, respectively, and in oat fiber, these values were 3.52 ml/g, 3.27 ml/g, and 3.4%, respectively, whereas in inulin, these values have been reported to be 0.08 ml/g, 2.53 ml/g, and 92.6%, respectively. Kurt and Kilincceker (96) studied the use of legume and cereal flours as fat replacers in beef patties. They substituted 5% of beef back fat with oat, rye, barley, rice, corn, wheat, soy, yellow lentil, and chickpea flours and evaluated the proximate composition of raw and cooked patties. Protein content increased in raw patties with oat, corn, soy, chickpea, and lentil flours but decreased in cooked patties with all cereal and legume flours except soy flour.

Moisture content did not change by the replacement of fat with the legume and cereal flours in both raw and cooked patties. Mehta et al. (108) reported the effects of psyllium on the proximate composition of the chicken patties, and the moisture, protein, fat, ash, TDF, SDF, and IDF contents of psyllium husk were 9.68%, 1.13%, 0.27%, 2.07%, 81.28%, 73.38%, and 7.91%, respectively. The level of psyllium husk influenced the product to a greater extent. Chicken patties with added psyllium husk at 4%, 6%, and 8% levels showed lower moisture and protein contents with increasing levels of psyllium husk. The researchers observed no significant difference in fat and ash contents at the different added psyllium husk levels.

Shobana et al. (138) reported that the total carbohydrates, dietary fiber, crude fiber, protein, crude fat, and total ash contents of finger millet flour were 72.0–79.5%, 18.6%, 3.7%, 7.0%, 1.3–1.8%, and 2.0–2.7% respectively. Chevon patties containing finger millet flour at levels 0%, 2%, 4%, and 6% were formulated by Kumar et al. (126). An increase in moisture, carbohydrate, and ash contents in cooked patties and a decrease in fat and protein contents were reported in both the raw and cooked patties with an increased amount of finger millet flour. Finger millet flour starch functioned as a water absorbent due to its hygroscopic nature, leading to more water retention in the patties. The mean carbohydrate and ash contents ranged from 7.69 to 7.81% and 2.83 to 3.05%, respectively.

*Algelica keiskei* is a rich source of dietary fiber (139) and contains various bioactive compounds such as saponins, germanium, coumarins, chalcones, and flavonoids (140). Choi et al. (125) stated that the moisture, protein, fat, ash, dietary fiber, and digestible carbohydrate contents of *A. keiskei* koidz powder were 7.93%, 16.54%, 5.03%, 11.93%, 4.23%, and 54.34%, respectively. Including *A. keiskei* Koidz in chicken patties also resulted in a significant increase in moisture and ash contents and a decrease in energy value compared to control products, but the protein content was unaffected.

Apple pomace is a major SDF source comprising pectin (141). The SDF content of apple pomace is mainly responsible for lowering the blood cholesterol level (142). The malic acid component of apples helps dissolve the lime deposits present in the body and lowers the incidence of fibrosis, arthritis, and rheumatism (143). Sun et al. (144) reported that the moisture and carbohydrate contents of apple pomace were 66.4–78.2% and 9.5–22.0%, respectively. Parkash et al. (134) studied the effects of dried apple pomace and corn bran on the proximate composition of chevon roll. They reported that moisture, protein, fat, ash, and crude fiber contents of dried carrot pomace were 4.11%, 2.81%, 4.16%, 1.84%, and 21.01%, respectively, and in corn bran, these values were 10.03%, 9.63%, 4.55%, 2.11%, and 17.07%, respectively. Corn bran at 3%, dried apple pomace at 6%, and corn bran + dried apple pomace at 2% + 3% levels were used separately in the chevon roll formulation. The moisture content decreased with the addition of dried apple pomace at 6% and corn bran + dried apple pomace at 2% + 3% levels. The protein content in chevon rolls showed a lower value with the addition of fibers. An increase in crude fiber content was reported in fiber-added chevon rolls.

Jabuticaba is a type of fruit, and its skin is rich in SDF (11.99 g/100 g dry matter) and IDF (26.43 g/100 g dry matter) (145). Jabuticaba skin flour has antimutagenic activity (146), reduces blood cholesterol levels in the blood (147), and can prevent obesity-associated insulin resistance (148). Weight loss in jabuticaba skin flour added restructured ham significantly increased by the flour levels (118). This increase in weight loss was attributed to decreased WHC of added fibers, resulting in the formation of exudates during cooking.

Carrot pomace has been used as a good source of insoluble dietary fibers in functional meat products. The major fiber-contributing constituents in carrot pomace are cellulose, hemicellulose, and pectic polysaccharides (149). Dried carrot pomace contains more dietary fiber due to loss of moisture. Yadav et al. (103) standardized the chicken sausages with wheat bran and dried carrot pomace. Protein, fat, and moisture contents decreased, whereas ash and crude fiber contents increased with the increased addition of wheat bran and dried carrot pomace. They attributed the decrease in moisture content to the relatively scanty water-binding capacity of the fibers used. The mean crude fiber value ranged from 0.56 to 1.28%. They reported that the TDF, IDF, SDF, and cholesterol contents of the 6% wheat bran incorporated chicken sausages were 2.98%, 2.76%, 0.22%, and 65.50% respectively. Whereas, in 6% of dried carrot pomace incorporated sausages, these values have been reported to be 3.77%, 3.32%, 0.45%, and 65.19%, respectively. An increase in both IDF and SDF in chicken sausages was observed. The cooking yield and ES of chicken sausages increased significantly at a 6% level of fiber incorporation. This improvement was assigned to an increase in water retention by the added wheat bran and dried carrot pomace.

### 4.3 Influence of dietary fiber on textural properties, color parameters, and sensory properties of meat products

Above all, the acceptability of meat products mostly depends on their textural characteristic, organoleptic properties, and associated color parameters. Adding fiber to meat products alters the texture, color, tenderness, flavor, and juiciness to a great extent. It has been observed that irrespective of the purpose of the addition of fibers, they enhance the functional properties and health benefits of meat products (150). The variation in these qualities is mostly influenced by the type of fiber (SDF/IDF), fiber source (fruits/vegetables/cereals/legumes, etc.), as well as the level of fiber added. The color of the developed product mostly depends on the color of the concerned fiber used and its inherent pigment sources. Turkish meatballs with four different levels (5%, 10%, 15%, and 20%) of corn, oat, and rye bran depicted higher yellowness value, and they attributed the increase in yellowness to higher carotenoid content in corn, rye, and oat bran (121). The lightness was increased and redness was decreased in Turkish meatballs with the addition of corn, oats, wheat, and rye bran. The lightness value was highest for 20% oat bran added to Turkish meatballs. Adding okara powder to the beef patties increased the yellowness and lightness value with a decrease in the redness value compared to the control product. The juiciness, tenderness, and overall acceptability of beef patties at more than 7.5% of incorporation of okara powder were significantly decreased compared to the control (97).

Mehta et al. (151) observed that the texture, flavor, color, and overall acceptability of chicken patties prepared with psyllium husk decreased with increased husk addition. The overall acceptability of the chicken patties decreased from 8.17% in control to 5.50% in psyllium husk (8%)-added chicken patties. The tenderness decreased as the husk content increased. They attributed the decrease in tenderness to the softening of products by incorporating a soluble dietary fiber. Huang et al. (133) observed no significant difference in lightness, redness, and yellowness of Chinese-style sausages at 3.5 and 7% levels of added wheat fiber, oat fiber, and inulin. The hardness value increased from 238.87 in control to 670.66 in 7% wheat fiber-added Chinese-style sausages. The hardness value increased from 238.87 in control to 457.82% in 7% oat fiber-added Chinese-style sausages. The sensory panel noted non-significant differences in hardness, cohesiveness, gumminess, and chewiness at different added insulin levels.

The effect of added rice bran and psyllium husk at the level of 10 + 2%, 10+ 4%, and 10 + 6%, respectively, on flavor, tenderness, juiciness, texture, color, and overall acceptability of patties and chicken meat rolls were assessed by Mehta et al. (108). The sensory score for all parameters decreased with increased incorporation levels of rice bran + psyllium husk combination. The control patties and chicken meat rolls had the highest sensory score values, whereas those with added 10% rice bran + 6% psyllium husk had the lowest sensory score values. The overall acceptability in the developed products remained far below the acceptable range at 10% rice bran + 6% psyllium husk incorporation. They concluded that 10% rice bran and 4% psyllium husk combination can be used in chicken meat rolls and patties formulation without adversely affecting the sensory qualities of the products.

The effect of added finger millet flour on the texture parameters of chevon patties was assessed by Kumar et al. (126). A texture profile analysis indicated decreased hardness, springiness, chewiness, stinginess, and gumminess of the chevon patties. An instrumental color profile analysis indicated that the lightness, yellowness, redness, and chroma value of the chevon patties decreased with increasing levels (2%, 4%, and 6%) of finger millet flour. Sensory scores of color or appearance, flavor, and overall acceptability of chevon patties with 4% finger millet flour showed no significant difference from those for chevon patties without finger millet flour. They concluded that 4% finger millet flour incorporation had higher overall acceptability, flavor, and sensory scores than the 6% level. Choi et al. (125) observed that the lightness and redness of both cooked and raw chicken patties prepared with *A. keiskei* Koidz fiber decreased with fiber addition. The lowest redness and lightness values were obtained for 4% *A. keiskei* Koidz dietary fiber among 0%, 1%, 2%, 3%, and 4% levels. The yellowness value increased as the *A. keiskei* Koidz fiber content increased. Gumminess, hardness, chewiness, and cohesiveness were decreased with increased *A. keiskei* Koidz fiber content in patties. The authors reported that the decrease in textural properties is possibly due to the loss of the fat and protein binding ability in the product and the higher WHC of the fibers.

Kim et al. (91) studied the effects of lessening fat levels (from 30 to 25, 20, and 15%) by replacing pork fat with pumpkin fiber (2%) and water in the frankfurters with respect to some sensory properties. An instrumental color analysis indicated that the lightness and redness scores of reduced-fat frankfurters with pumpkin fiber (2%) were lower than the frankfurter with 30% fat, and the yellowness score was higher in the frankfurters with added pumpkin fiber (2%) than the products without pumpkin fiber and highest yellowness value in frankfurters having 15% fat and 2% added fiber of pumpkin. A texture profile analysis concluded that the hardness of frankfurters with pumpkin fiber was higher than reduced-fat frankfurters without pumpkin fiber and frankfurters with 30% added fat. Gumminess, cohesiveness, and chewiness of frankfurters with pumpkin fiber (2%) decreased with increased fat replacement levels with added water. The flavor, texture, tenderness, juiciness, and overall acceptability scores of chicken sausages at a 6% level of incorporation of corn bran, dried apple pomace, and dried tomato pomace significantly decreased as compared to the control (115). In addition, the sensory properties of goat meat nuggets at 5% incorporation of aloe vera gel were significantly affected compared to the control (112).

The effect of the addition of dried carrot pomace and wheat bran at the levels of 3%, 6%, and 9% separately on shear press value, cohesiveness, hardness, gumminess, chewiness, springiness, and color properties of chicken sausages were assessed by Yadav et al. (103). Chicken sausages with either wheat bran or dried carrot pomace were observed as having a harder texture than those without wheat bran or dried carrot pomace. Hardness value increased as wheat bran and dried carrot pomace contents increased. Springiness and cohesiveness of chicken sausages were gradually decreased with increased levels of dried carrot pomace and wheat bran from 3 to 9%. They attributed the increase to a complex network structure in the meat matrix due to higher insoluble fiber content in chicken sausage, as fiber generally exhibits high water-binding capacity. Similar observations regarding higher wheat bran and carrot dietary fiber resulting in a complex network structure leading to a harder texture and higher water-binding capacity have been reported (152, 153). Gumminess and chewiness of chicken sausages gradually increased as the level of wheat bran increased, the values decreased with increased dried carrot pomace level, and the values were lowest at 9% dried carrot pomace in the product. Results showed that the chicken sausages with higher levels of wheat bran required a higher shear press to break them. Decreased shear press value was observed with the incorporation of dried carrot pomace. The sensory panel reported non-significant differences in lightness and redness at the different added fiber levels. Yellowness increased with wheat bran addition. They attributed the increase in yellowness to the diversified colors and the presence of carotenoid pigments in the fibers. Similar observations with respect to higher carotenoid content leading to increased yellowness value have been reported (121). The effect of dietary fiber on texture, color, and sensory properties of processed meat products has been critically reviewed and presented in Table 3.

**Table 3:** Influence of dietary fiber on textural properties, color parameters, and sensory properties of meat products.

**Source of dietary fiber**	**Meat product**	**Optimum level of incorporation by replacing meat**	**Changes in attributes**	**References**
Corn, oats, and rye bran	Turkish meatballs	10%	Higher yellowness value Lightness increased and redness decreased	(121)
Okara powder	Beef patties	7.5%	Increased the yellowness and lightness value with a decrease in the redness value	(97)
Inulin	Chinese-style sausages	3.5%	No significant difference in lightness, redness, yellowness, hardness, cohesiveness, gumminess, and chewiness value	(133)
Psyllium husk and black gram hull	Chicken patties	4% psyllium husk+ 5% black gram hull	Color, juiciness, tenderness, flavor, texture, and overall acceptability score decreased	(122)
Citrus fiber	Low-fat frankfurters	2%	Hardness increased	(106)
Finger millet flour	Chevon patties	4%	Hardness, springiness, chewiness, stinginess, gumminess, lightness, yellowness, redness, and chroma value decreased	(126)
*Algelica keiskei* koidz	Chicken patties	2%	Gumminess, hardness, chewiness, cohesiveness lightness and redness values decreased	(125)
Pumpkin fiber	Pork frankfurters	2%	Lightness and redness scores decreased, hardness increased Gumminess, cohesiveness, and chewiness decreased	(91)
Gum arabic	Beef patties	1.5%	Enhanced the lightness and yellowness value in beef patties	(120)
Aloe vera gel	Goat meat nuggets	2.5%	Hardness, fracturability, adhesiveness, chewiness, and shear force value decreased	(112)
Lotus rhizome powder	Sausages	3%	Springiness increased No significant difference in cooked meat flavor, overall acceptability Hardness, cooked meat color, lightness and redness values decreased	(119)
Inulin	Sausages	6%	Decreased cohesiveness no significant difference in springiness, gumminess, chewiness, and color parameters	(30)
Dried carrot pomace	Chicken sausages	6%	Hardness value increased Springiness and cohesiveness decreased non-significant difference in lightness and redness value	(103)
Inulin	Chicken meatballs	3%	Color values increased	(93)
Dried kinnow pomace powder	Pork patties	4%	Hardness, chewiness, and gumminess increased Lightness, yellowness, redness, and overall acceptability decreased	(124)
Rice bran	Hamburger	4%	Lightness and redness values decreased and yellowness value increased	(31)
Chia flour	Chicken nuggets	10%	Cohesiveness, springiness, lightness, yellowness value, and redness value increased	(113)

### 4.4 Effect of the addition of dietary fiber on the fatty acid composition of the meat products

Dietary fibers from diverse sources contain different fatty acid compositions and accordingly alter the composition of fatty acid of the developed product upon addition. Yilmaz (111) observed that the saturated fatty acid (SFA) content decreased and total unsaturated fatty acid (USFA) content of meatballs increased significantly with an increase in the level of rye bran in the product. Hu and Yu (94) studied the addition of hemicellulose B (from defatted rice bran) into meatballs at three different levels (2%, 4%, and 6%). They noted a higher ratio of total USFA content to total SFA content and a lower fat content and trans fatty acid content in hemicellulose B added meatballs than in the control products. Rajkumar et al. (112) observed an increased monounsaturated fatty acid content and a decreased SFA content in the aloe vera gel-added goat meat nuggets. Barros et al. (113) utilized chia flour in chicken nuggets and reported an increased α-linolenic and decreased oleic acid, 7-hexadecenoic acid, and paullinic acid contents in chia flour-added nuggets. The authors also reported that the content of polyunsaturated fatty acid (PUFA) increased significantly in chia flour-added chicken nuggets. The omega 6:omega 3 ratio decreased and the PUFA:SFA ratio increased in chia flour added to chicken nuggets.

## 5 Conclusion

The techno-functional and health-promoting properties of dietary fiber can be used effectively in the development of fiber-enriched meat products, which will certainly obviate the negative perception of consumers about red meat (154–174). The positive physiological effects of dietary fibers on the control and prevention of lifestyle diseases such as cardiovascular diseases, various cancers, diabetes, and obesity have been documented. The addition of fibers significantly influences the cooking yield, ES, WHC, juiciness, color, and texture of the meat products. Also, fibers can be used as a fat replacer in the meat products. At different times, various meat scientists have identified potential sources of dietary fiber for their inclusion in meat products to develop more nutritious, healthier, and functional products with acceptable organoleptic properties and proven efficiency. The imperative of our time lies in the advancement of novel meat products fortified with dietary fiber to address the escalating prevalence of lifestyle-related medical conditions. Further investigation can be conducted to examine the prospective origins of dietary fibers that possess heightened bioactive compounds to advance the production of functional meat products.

## Author contributions

BM: Conceptualization, Data curation, Formal analysis, Funding acquisition, Investigation, Resources, Supervision, Visualization, Writing – original draft, Writing – review & editing. JM: Formal analysis, Investigation, Visualization, Writing – review & editing. BP: Conceptualization, Formal analysis, Resources, Supervision, Validation, Writing – original draft, Writing – review & editing. PR: Data curation, Formal analysis, Visualization, Writing – review & editing. MJ: Data curation, Formal analysis, Visualization, Writing – review & editing. BR: Data curation, Formal analysis, Visualization, Writing – review & editing. PP: Data curation, Formal analysis, Visualization, Writing – review & editing. SP: Data curation, Formal analysis, Visualization, Writing – review & editing. DS: Supervision, Visualization, Writing – review & editing, Formal analysis, Investigation.
